# Chronic N-Acetyl-Cysteine Treatment Enhances the Expression of the Immediate Early Gene *Nr4a1* in Response to an Acute Challenge in Male Rats: Comparison with the Antidepressant Venlafaxine

**DOI:** 10.3390/ijms24087321

**Published:** 2023-04-15

**Authors:** Paola Brivio, Maria Teresa Gallo, Piotr Gruca, Magdalena Lason, Ewa Litwa, Fabio Fumagalli, Mariusz Papp, Francesca Calabrese

**Affiliations:** 1 Department of Pharmacological and Biomolecular Sciences “Rodolfo Paoletti”, Università degli Studi di Milano, 20133 Milan, Italy; 2 Maj Institute of Pharmacology, Polish Academy of Sciences, 31-343 Krakow, Poland

**Keywords:** restraint, antioxidant, IEGs, neuroplastic mechanisms, rats, psychiatric disorders

## Abstract

Despite several antidepressant treatments being available in clinics, they are not effective in all patients. In recent years, N-acetylcysteine (NAC) has been explored as adjunctive therapy for many psychiatric disorders, including depression, for its antioxidant properties. Given the promising efficacy of this compound for the treatment of such pathologies, it is fundamental to investigate, at the preclinical level, the ability of the drug to act in the modulation of neuroplastic mechanisms in basal conditions and during challenging events in order to highlight the potential features of the drug useful for clinical efficacy. To this aim, adult male Wistar rats were treated with the antidepressant venlafaxine (VLX) (10 mg/kg) or NAC (300 mg/kg) for 21 days and then subjected to 1 h of acute restraint stress (ARS). We found that NAC enhanced the expression of several immediate early genes, markers of neuronal plasticity in the ventral and dorsal hippocampus, prefrontal cortex and amygdala, and in particular it mediated the acute-stress-induced upregulation of *Nr4a1* expression more than VLX. These data suggested the ability of NAC to induce coping strategies to face external challenges, highlighting its potential for the improvement of neuroplastic mechanisms for the promotion of resilience, in particular via the modulation of *Nr4a1*.

## 1. Introduction

Despite depression being one of the largest causes of disease burden globally [1], the currently available antidepressants show several limitations, as they exhibit a low rate of full remission, residual symptoms after treatment and a high relapse rate [2,3]. Moreover, the lack of a clear biological pathogenesis of depression results in a latency in their effectiveness, making it difficult to develop new antidepressants. These clinical unmet needs have pushed research toward the development of adjuvants that may boost the action of antidepressants.

N-acetylcysteine (NAC) is a molecule used as a mucolytic for chronic obstructive pulmonary disease, as a protector for contrast-related nephropathy, as an agent for the management of HIV and as an antidote in the treatment of paracetamol overdose [4]. NAC is a substrate for glutathione synthesis and is believed to exert antioxidant properties at multiple levels, since glutathione is a ubiquitous intracellular antioxidant that regulates oxidative homeostasis in the cells and acts as scavenger of reactive oxidative species (ROS).

Besides the treatment of the above-mentioned pathological states, NAC has been reported to positively interfere with several pathophysiological processes in depression when administered as a supplementation of pharmacological therapy [5] and in other mental disorders [6], since its mechanism of action overlaps with the functional changes associated with these mental illnesses. Indeed, redox dysregulation also appears to have a significant role in the development of psychiatric diseases [7].

Hence, deepening the understanding of the mechanism of action of NAC in experimental models is critical to improve the knowledge on this potential novel therapy for depression and psychiatric disorders.

In this study, we aimed to establish whether chronic NAC administration may influence the responsiveness to a novel acute challenge in comparison to the antidepressant venlafaxine (VLX), one of the most efficient treatments for depression [8]. To this purpose, adult male rats, previously exposed to 21 days of intraperitoneal administration with VLX or NAC, were subjected to one hour of acute restraint stress (ARS). Acute stress can cause adaptations in the brain through dynamic changes in the expression of the immediate early genes (IEGs), while repeated stimulation can have a repressive effect on it [9]. The study of IEG expression, a well-established index of neuronal activation, is a proxy of the brain’s ability to properly react to both positive and negative external cues and their rapid transcription is required for critical brain function, including cognitive behavior [10].

Accordingly, we previously demonstrated that acute restraint stress improved the performance in a cognitive test by enhancing the expression of different IEGs [11], while chronic stress interfered with the ability to react to a subsequent acute challenge in vulnerable but not resilient rats in a brain-region-specific manner [12,13]. Moreover, we observed that antidepressant and antipsychotic treatments positively modulated the response to another type of acute stressor, the forced swim stress [14,15,16,17], suggesting that such treatments can help with coping with stressors of different natures.

Here, we investigated the potential ability of rats to adopt active strategies by evaluating the enhancement of the IEGs *Arc*, *c-Fos*, *Gadd45β*, *Npas4*, *Nr4a1* and *Zif268* to cope with the ARS following chronic VLX and NAC administration. We examined the activation/implication of different brain regions, namely, the ventral and dorsal hippocampus and the prefrontal cortex and amygdala, which are relevant areas for the activity of the IEGs [18] and are implicated in depression and psychiatric disorders. Our results suggest that NAC pretreatment positively modulates the expression of the IEGs that contribute to facing the novel challenge, at least in the brain areas investigated. In addition, it appears that NAC pretreatment increased the expression of *Nr4a1* more than that observed with the antidepressant VLX.

## 2. Results

### 2.1. Chronic NAC Treatment Enhanced c-Fos and Nr4a1 Gene Expression More Than VLX in the Ventral Hippocampus of Acutely Stressed Rats

**Figure 1:**
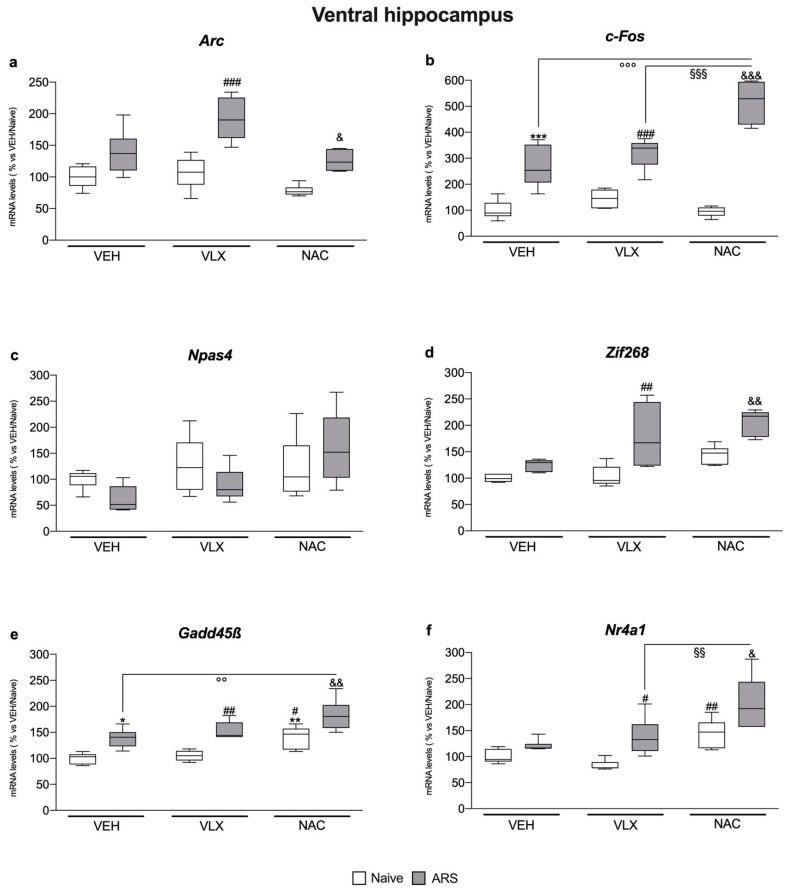
Analysis of *Arc* (**a**), *c-Fos* (**b**), *Npas4* (**c**), *Zif268* (**d**), *Gadd45β* (**e**), and *Nr4a1* (**f**) mRNA levels in the ventral hippocampus of rats treated with VLX (10 mg/kg) or NAC (300 mg/kg) and exposed to 1 h of acute restraint stress (ARS). The data are the mean ± SEM of 6 independent determinations. * *p* < 0.05, ** *p* < 0.01, *** *p* < 0.001 vs. VEH/Naïve; ^#^ *p* < 0.05, ^##^ *p* < 0.01, ^###^ *p* < 0.001 vs. VLX/Naïve; ^&^ *p* < 0.05, ^&&^ *p* < 0.01, ^&&&^ *p* < 0.001 vs. NAC/Naïve; ^°°^ *p* < 0.01, ^°°°^ *p* < 0.001 vs. VEH/ARS; ^§§^ *p* < 0.01, ^§§§^ *p* < 0.001 vs. VLX/ARS. (Two-way ANOVA followed by Tukey’s multiple comparisons test).

In the ventral hippocampus, we observed that, independently from the pretreatment, 1 h of ARS induced a significant overexpression of *c-Fos* (F_1,32_ = 173, two-way ANOVA; VEH: +168%, *p* < 0.001 vs. VEH/Naïve; VLX: +122%, *p* < 0.001 vs. VLX/Naïve; NAC: +451%, *p* < 0.001 vs. NAC/Naïve, Tukey multiple comparison) and *Gadd45β* (F_1,34_ = 45.2, two-way ANOVA; +39%, *p* < 0.05 vs. VEH/Naïve; VLX: +45%, *p* < 0.01 vs. VLX/Naïve; NAC: +30%, *p* < 0.01 vs. NAC/Naïve, Tukey multiple comparison) when compared to their naïve counterpart (Figure 1b–e).

Moreover, as shown in Figure 1a,d,f, ARS increased the mRNA levels of *Arc* (F_1,34_ = 49.5 two-way ANOVA; VLX: +82%, *p* < 0.001 vs. VLX/Naïve; NAC: +61%, *p* < 0.05 vs. NAC/Naïve, Tukey multiple comparison), *Zif268* (F_1,33_ = 34.8, two-way ANOVA; VLX: +73%, *p* < 0.01 vs. VLX/Naïve; NAC: +43%, *p* < 0.01 vs. NAC/Naïve, Tukey multiple comparison) and *Nr4a1* (F_1,35_ = 22.1, two-way ANOVA; VLX: +66%, *p* < 0.05 vs. VLX/Naïve; NAC: +40%, *p* < 0.05 vs. NAC/Naïve, Tukey multiple comparison), in comparison with the respective naïve group, only in rats chronically pre-treated with VLX and NAC but not in the group pre-exposed to the vehicle.

Interestingly, the increase in gene expression due to NAC administration in response to the acute stress was higher than that exerted by VLX for the gene expression of *c-Fos* (F_2,31_ = 17.5, two-way ANOVA; +61%, *p* < 0.001 vs. VLX/ARS, Tukey multiple comparison) (Figure 1b) and *Nr4a1* (F_2,34_ = 1.57, two-way ANOVA; +46%, *p* < 0.01 vs. VLX/ARS, Tukey multiple comparison) (Figure 1f), and also greater than the effect observed in vehicle-treated animals for *c-Fos* (+93%, *p* < 0.001 vs. VEH/ARS, Tukey multiple comparison) and *Gadd45β* (F_2,33_ = 0.16, two-way ANOVA; +32%, *p* < 0.01 vs. VEH/ARS, Tukey multiple comparison).

Finally, we observed that *Gadd45β* mRNA levels (Figure 1e) were increased by NAC administration in the basal condition with respect to the VEH/Naïve (F_2,33_ = 16.7, two-way ANOVA; +41%, *p* < 0.01 vs. VEH/Naïve, Tukey multiple comparison) and VLX/Naïve groups (+34%, *p* < 0.05 vs. VLX/Naive, Tukey multiple comparison), whereas *Nr4a1* gene expression (Figure 1f) was upregulated by NAC versus VLX/Naïve animals (F_2,34_ = 16.9, two-way ANOVA; +74%, *p* < 0.01 vs. VLX/Naïve, Tukey multiple comparison).

### 2.2. IEG Expression in Dorsal Hippocampus Was Differently Modulated by ARS and the Treatments: Comparison with Ventral Hippocampus

In the dorsal hippocampus, we observed a different modulation exerted by ARS and by treatments on IEG expression in comparison to ventral hippocampus.

**Figure 2:**
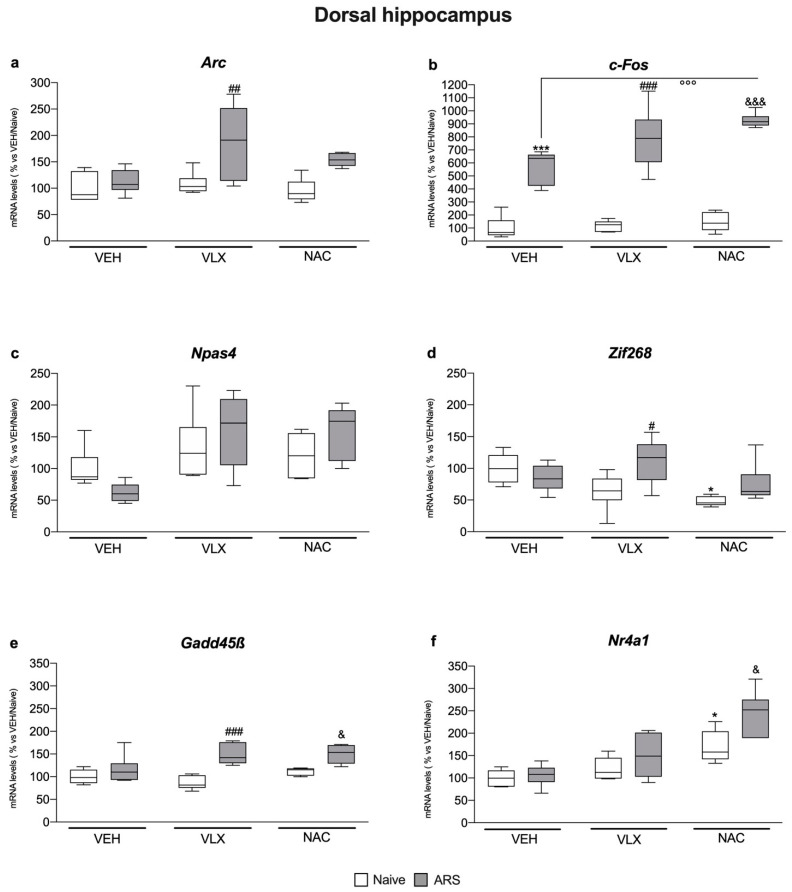
Analysis of *Arc* (**a**), *c-Fos* (**b**), *Npas4* (**c**), *Zif268* (**d**), *Gadd45β* (**e**), and *Nr4a1* (**f**) mRNA levels in the dorsal hippocampus of rats treated with VLX or NAC and exposed to 1 h of acute restraint stress (ARS). The data are the mean ± SEM of 6 independent determinations. * *p* < 0.05, *** *p* < 0.001 vs. VEH/Naïve; ^#^ *p* < 0.05, ^##^ *p* < 0.01, ^###^ *p* < 0.001 vs. VLX/Naïve; ^&^ *p* < 0.05, ^&&&^ *p* < 0.001 vs. NAC/Naïve; ^°°°^ *p* < 0.001 vs. VEH/ARS. (Two-way ANOVA followed by Tukey’s multiple comparisons test).

We observed that only *c-Fos* gene expression was upregulated by the ARS independently from the pretreatments (F_1,34_ = 244, two-way ANOVA; VEH: +473%, *p* < 0.001 vs. VEH/Naïve; VLX: +545%, *p* < 0.001 vs. VLX/Naïve; NAC: +536%, *p* < 0.001 vs. NAC/Naïve, Tukey multiple comparison) (Figure 2b).

Moreover, acute stress increased mRNA levels of *Arc* (F_2,34_ = 2.99, two-way ANOVA; +73%, *p* < 0.01 vs. VLX/Naïve, Tukey multiple comparison), *Zif268* (F_2,33_ = 4.52, two-way ANOVA; +75%, *p* < 0.05 vs. VLX/Naïve, Tukey multiple comparison) and *Gadd45β* (F_2,34_ = 4.12, two-way ANOVA; +73%, *p* < 0.001 vs. VLX/Naïve, Tukey multiple comparison) in animals pretreated with VLX (Figure 2a,d,e), and of *Gadd45β* (+34%, *p* < 0.05 vs. NAC/Naïve, Tukey multiple comparison) and *Nr4a1* (F_2,34_ = 2.80, two-way ANOVA; +44%, *p* < 0.05 vs. NAC/Naïve, Tukey multiple comparison) in rats previously administered with NAC (Figure 2e,f).

Further, the effect of NAC pretreatment on the response to ARS was higher than that found in rats treated with VEH for *c-Fos* (F_2,33_ = 4.97, two-way ANOVA; +62%, *p* < 0.001 vs. VEH/ARS, Tukey multiple comparison) (Figure 2b).

Of note, as shown in Figure 2d,f, NAC administration decreased the expression of *Zif268* (F_2,33_ = 4.92, two-way ANOVA; −52%, *p* < 0.05 vs. VEH/Naïve, Tukey multiple comparison) and increased the gene expression of *Nr4a1* (F_2,34_ = 26.4, two-way ANOVA; +70%, *p* < 0.05 vs. VEH/Naïve, Tukey multiple comparison) with respect to vehicle-treated rats.

### 2.3. In the Prefrontal Cortex, Acute Stress Modulated IEG Expression Independently from Pretreatments

**Figure 3:**
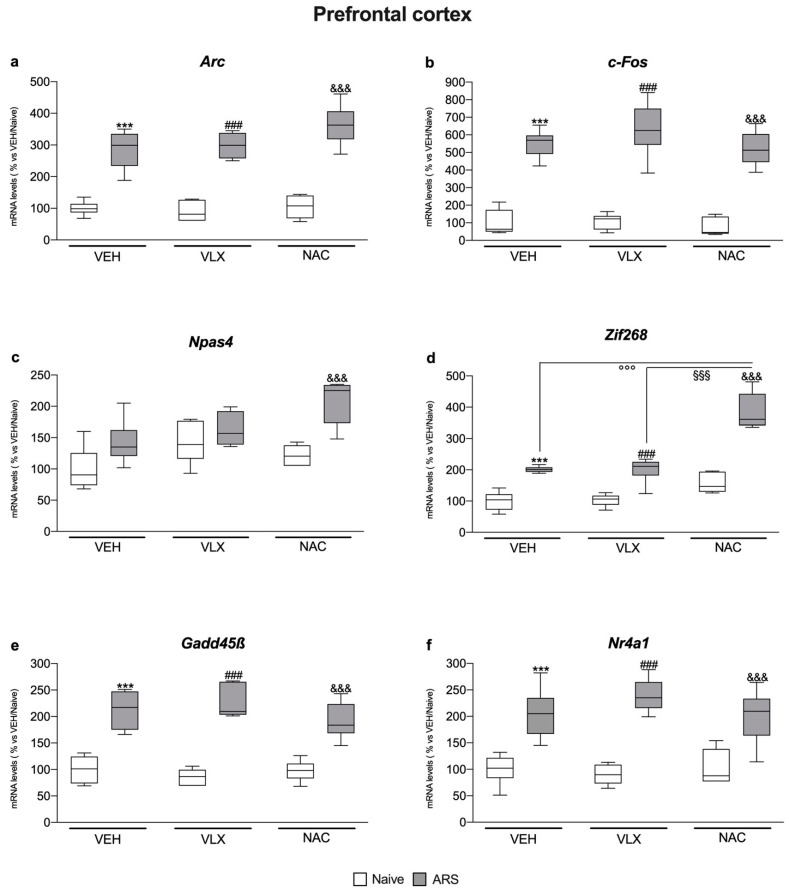
Analysis of *Arc* (**a**), *c-Fos* (**b**), *Npas4* (**c**), *Zif268* (**d**), *Gadd45β* (**e**), and *Nr4a1* (**f**) mRNA levels in the prefrontal cortex of rats treated with VLX or NAC and exposed to 1 h of acute restraint stress (ARS). The data are the mean ± SEM of 6 independent determinations. *** *p* < 0.001 vs. VEH/Naive; ^###^ *p* < 0.001 vs. VLX/Naïve; ^&&&^ *p* < 0.001 vs. NAC/Naïve; ^°°°^ *p* < 0.001 vs. VEH/ARS; ^§§§^ *p* < 0.001 vs. VLX/ARS. (Two-way ANOVA followed by Tukey’s multiple comparisons test).

In the prefrontal cortex we observed that, independently from the pretreatments, ARS induced an overexpression of *Arc* (F_1,35_ = 210, two-way ANOVA; VEH: +186%, *p* < 0.001 vs. VEH/Naïve; VLX: +231%, *p* < 0.001 vs. VLX/Naïve; NAC: +247%, *p* < 0.001 vs. NAC/Naïve, Tukey multiple comparison), *c-Fos* (F_1,34_ = 235, two-way ANOVA; VEH: +451%, *p* < 0.001 vs. VEH/Naïve; VLX: +482%, *p* < 0.001 vs. VLX/Naïve; NAC: +567%, *p* < 0.001 vs. NAC/Naïve, Tukey multiple comparison), *Zif268* (F_1,34_ = 155, two-way ANOVA; VEH: +101%, *p* < 0.001 vs. VEH/Naïve; VLX: +94%, *p* < 0.001 vs. VLX/Naïve; NAC: +146%, *p* < 0.001 vs. NAC/Naïve, Tukey multiple comparison), *Gadd45β* (F_1,35_ = 158, two-way ANOVA; VEH: +112%, *p* < 0.001 vs. VEH/Naïve; VLX: +164%, *p* < 0.001 vs. VLX/Naïve; NAC: +97%, *p* < 0.001 vs. NAC/Naïve, Tukey multiple comparison) and *Nr4a1* (F_1,35_ = 94.3, two-way ANOVA; VEH: +105%, *p* < 0.001 vs. VEH/Naïve; VLX: +167%, *p* < 0.001 vs. VLX/Naïve; NAC: +95%, *p* < 0.001 vs. NAC/Naïve, Tukey multiple comparison) (Figure 3a,b,d,e,f).

Moreover, as shown in Figure 3c, ARS increased the gene expression of *Npas4* specifically in rats previously administered with NAC (F_2,33_ = 3.26, two-way ANOVA; +71%, *p* < 0.001 vs. NAC/Naïve, Tukey multiple comparison) with respect to their naïve counterpart.

In addition, the increase due to ARS was higher in animals pretreated with NAC with respect to *Zif268* (Figure 3d), not only versus the VEH/ARS group (F_2,33_ = 13.8, two-way ANOVA; VEH: +92%, *p* < 0.001 vs. VEH/ARS, Tukey multiple comparison) but also in comparison to the VLX/ARS animals (VEH: +92%, *p* < 0.001 vs. VEH/ARS, Tukey multiple comparison).

### 2.4. Chronic NAC Treatment Induced Higher IEG Expression in Comparison to VLX in the Amygdala of Acutely Stressed Rats

**Figure 4:**
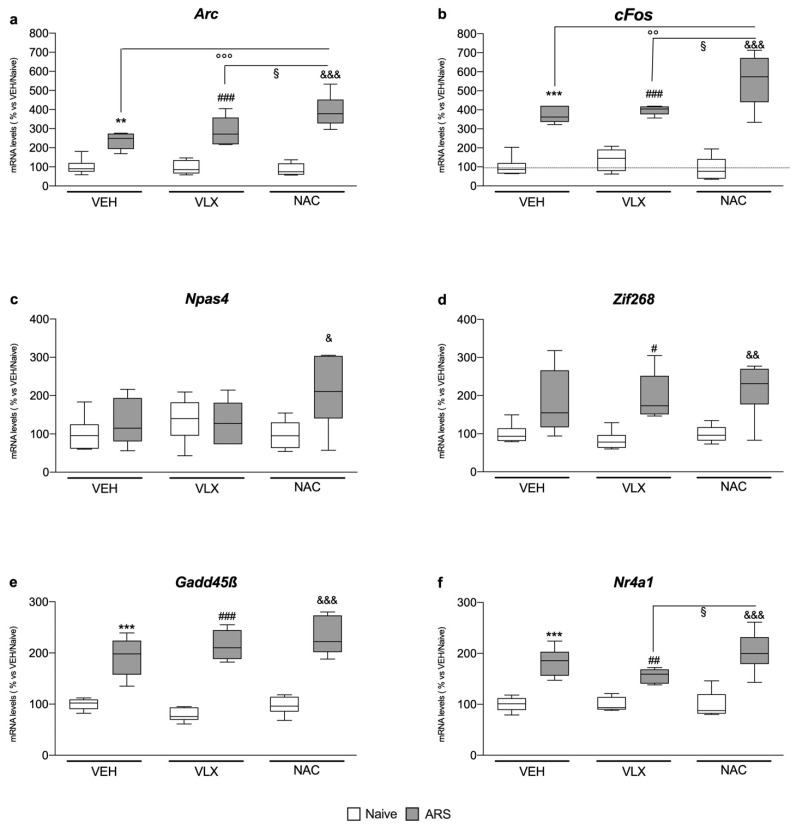
Analysis of *Arc* (**a**), *c-Fos* (**b**), *Npas4* (**c**), *Zif268* (**d**), *Gadd45β* (**e**), and *Nr4a1* (**f**) mRNA levels in the amygdala of rats treated with VLX or NAC and exposed to 1 h of acute restraint stress (ARS). The data are the mean ± SEM of 6 independent determinations. ** *p* < 0.01, *** *p* < 0.001 vs. VEH/Naïve; ^#^ *p* < 0.05, ^##^ *p* < 0.01, ^###^ *p* < 0.001 vs. VLX/Naïve; ^&^ *p* < 0.05, ^&&^ *p* < 0.01, ^&&&^ *p* < 0.001 vs. NAC/Naïve; ^°°^ *p* < 0.01, ^°°°^ *p* < 0.001 vs. VEH/ARS; ^§^ *p* < 0.05 vs. VLX/ARS. (Two-way ANOVA followed by Tukey’s multiple comparisons test).

In the amygdala, we found that ARS, independently from the treatments, increased the expression of *Arc* (F_1,35_ = 129, two-way ANOVA; VEH: +136%, *p* < 0.01 vs. VEH/Naïve; VLX: +200%, *p* < 0.001 vs. VLX/Naïve; NAC: +355%, *p* < 0.001 vs. NAC/Naïve, Tukey multiple comparison), *c-Fos* (F_1,33_ = 170, two-way ANOVA; VEH: +275%, *p* < 0.001 vs. VEH/Naïve; VLX: +189%, *p* < 0.001 vs. VLX/Naïve; NAC: +510%, *p* < 0.001 vs. NAC/Naïve, Tukey multiple comparison), *Gadd45β* (F_1,35_ = 186, two-way ANOVA; VEH: +92%, *p* < 0.001 vs. VEH/Naïve; VLX: +174%, *p* < 0.001 vs. VLX/Naïve; NAC: +139%, *p* < 0.001 vs. NAC/Naïve, Tukey multiple comparison) and *Nr4a1* (F_1,34_ = 93.7, two-way ANOVA; VEH: +83%, *p* < 0.001 vs. VEH/Naïve; VLX: +56%, *p* < 0.01 vs. VLX/Naïve; NAC: +105%, *p* < 0.001 vs. NAC/Naïve, Tukey multiple comparison) (Figure 4a,b,e,f).

Moreover, NAC pretreatment in acutely stressed animals induced a greater upregulation of the mRNA levels of *Arc* and *c-Fos* than the one observed in the VEH/ARS group (*Arc*: F_2,34_ =7.18, two-way ANOVA; +65%, *p* < 0.001 vs. VEH/ARS; *c-Fos*: F_2,32_ = 6.77, two-way ANOVA; +48%, *p* < 0.01 vs. VEH/ARS, Tukey multiple comparison) and in the VLX/ARS animals (*Arc*: +36%, *p* < 0.05 vs. VLX/ARS; *c-Fos*: +39%, *p* < 0.05 vs. VLX/ARS, Tukey multiple comparison), and of *Nr4a1* specifically versus stressed animals administered with VLX (F_2,33_ = 2.72, two-way ANOVA; +60%, *p* < 0.05 vs. VLX/ARS, Tukey multiple comparison).

Furthermore, as shown in Figure 4c,d, VLX pretreatment induced increased mRNA levels of *Zif268* following the ARS (F_2,34_ = 0.38, two-way ANOVA; +139%, *p* < 0.05 vs. VLX/Naïve, Tukey multiple comparison,) whereas NAC administration increased both *Npas4* (F_2,33_ = 2.82, two-way ANOVA; +114%, *p* < 0.05 vs. NAC/Naïve, Tukey multiple comparison) and *Zif268* (+118%, *p* < 0.01 vs. NAC/Naïve, Tukey multiple comparison) gene expression in acutely stressed animals with respect to their naïve counterpart.

### 2.5. Z-Score Activation Underlines the Role of NAC in Enhancing Nr4a1 Gene Expression Specifically

**Figure 5:**
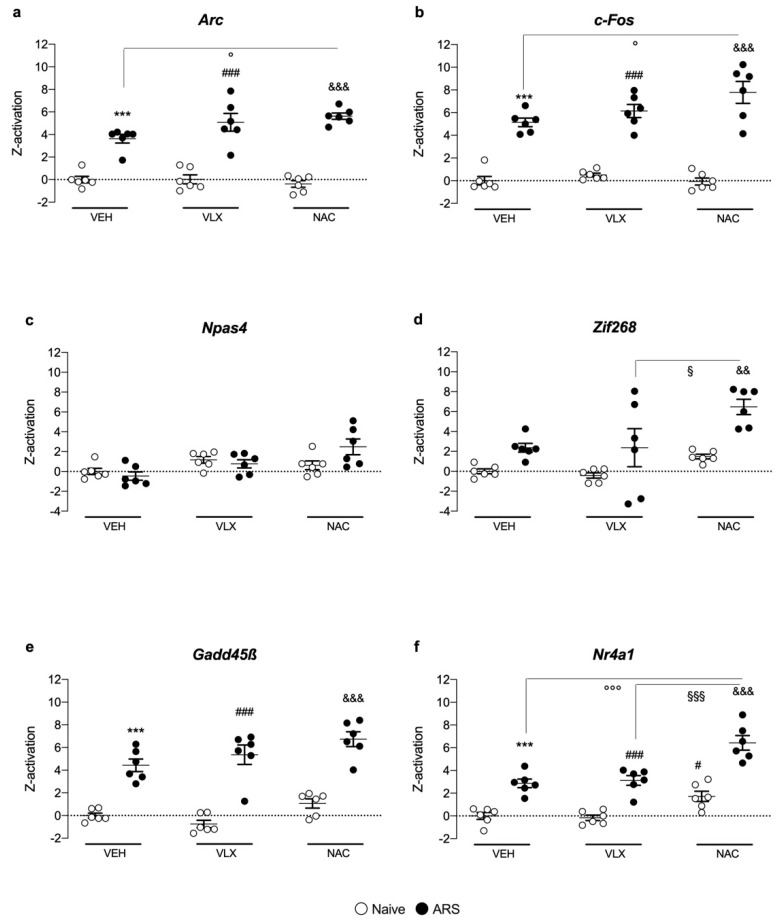
Z-score activation of *Arc* (**a**), *c*-*Fos* (**b**), *Npas4* (**c**), *Zif268* (**d**), *Gadd45β* (**e**), and *Nr4a1* (**f**) in the ventral hippocampus, dorsal hippocampus, prefrontal cortex and amygdala of rats pretreated with VLX or NAC and exposed to 1 h of acute restraint stress (ARS). Data are expressed as mean ± SEM. *** *p* < 0.001 vs. VEH/Naïve; ^#^ *p* < 0.05, ^###^ *p* < 0.001 vs. VLX/Naïve; ^&&^ *p* < 0.01 ^&&&^ *p* < 0.001 vs. NAC/Naïve; ^°^ *p* < 0.05, ^°°°^ *p* < 0.001 vs. VEH/ARS; ^§^ *p* < 0.05, ^§§§^ *p* < 0.001 vs. VLX/ARS. (Two-way ANOVA followed by Tukey’s multiple comparisons test).

As shown in Figure 5, we calculated the Z-score activation of each target gene in all the brain regions to obtain a combined overview of the modulation of the six immediate early genes previously analyzed. We found a significant increase in the Z-score activation of *Arc* (F_1,35_ = 183, two-way ANOVA), *c-Fos* (F_1,35_ = 207, two-way ANOVA), *Gadd45β* (F_1,35_ = 147, two-way ANOVA) and *Nr4a1* (F_1,35_ = 109, two-way ANOVA) due to stress exposure in all the brain regions examined, regardless of the pretreatment.

Moreover, we observed that NAC pretreatment in acutely stressed rats induced an overall increase in *Arc* (F_2,34_ = 3.67, two-way ANOVA), *c-Fos* (F_2,34_ = 3.71, two-way ANOVA) and *Nr4a1* (F_2,33_ = 2.59, two-way ANOVA) with respect to the VEH/ARS group.

Further, as a matter of fact, specifically for *Nr4a1*, we found that rats pretreated with NAC and subjected to ARS showed a Z-activation that was significantly higher than that related not only to the VEH group but also to the VLX group.

## 3. Discussion

Our findings show that pretreatments with NAC and VLX modulate the ARS-induced response through the regulation of several IEGs in a brain-region-specific manner. In particular, NAC/VLX pretreatments influenced the response to ARS primarily in the ventral hippocampus and in the amygdala.

We have previously shown that the antidepressants duloxetine [17] and vortioxetine [14] and the antipsychotics lurasidone [16] and blonanserine [15] promoted IEG expression while handling an acute challenge, suggesting that IEG modulation may represent a mechanism common to antidepressant and antipsychotic drugs in orchestrating an adequate response to an acute stressful event.

Examining the overall effects of NAC or VLX in the different brain regions, we found that the most consistent effects of NAC pretreatment on the response to the ARS, in comparison to the modulation exerted by VLX, were observed in the ventral hippocampus and amygdala, two limbic structures critical for the processing of external stimuli, the gating of behavioral responses, the regulation of emotional responses and the orchestration of memory storage [19,20]. Specifically, we observed that *c-Fos* and *Nr4a1* in the ventral hippocampus and *c-Fos*, *Nr4a1* and *Arc* in the amygdala were extremely sensitive to NAC pretreatment in the response to the acute challenge.

Conversely, we depicted a different situation in the prefrontal cortex, where we found that the acute challenge indeed modulated IEGs expression but regardless of the pretreatments, and in the dorsal hippocampus, where a more heterogeneous pattern of IEGs activation was observed.

This complex scenario prompted us to calculate the Z-activation, as a comprehensive level of analysis, for each target gene across the ventral and dorsal hippocampus, prefrontal cortex and amygdala. The mathematical tool predicted that *Nr4a1* was the IEG with the strongest modulation by NAC following ARS versus VLX stressed animals, suggesting that NAC promotes *Nr4a1* gene expression to favor coping with the acute challenge more than the classical antidepressant VLX.

*Nr4a1* is an activity-dependent transcription factor rapidly transcribed in response to neuronal activity and stress that plays a fundamental role in the mechanisms that regulate the adaptive abilities of the brain, ranging from synaptic plasticity and dendritic remodeling [21,22] to energy homeostasis and mitochondrial function [23,24,25]. In addition, it is a critical mediator of long-term memory [26,27,28], a process that requires neuronal activity via the transcriptional activation of the IEGs [29], which results in the expression of critical genes for memory consolidation [10]. Thus the NAC-mediated enhancement of *Nr4a1* in response to the ARS suggests the possibility that this compound could be useful in modulating the signaling cascade regulated by the rapid *Nr4a1* transcription, hence favoring long-term synaptic changes [30]. Furthermore, it has been observed that *Nr4a1* mediates Gpx1 expression by binding the putative binding site on the Gpx1 promoter [31], strengthening our finding that NAC, a natural precursor of Gpx1, mediated the coping with a novel challenge by activating *Nr4a1* expression primarily. Moreover, in addition to its classification as an IEG, *Nr4a1* contributes to the activation of apoptosis via mitochondria [32] in response to negative external stimuli. Indeed, *Nr4a1* can exert opposing biological activities depending on its subcellar distribution and its transient expression. Considering this aspect, it follows that our results suggest that the modulation of *Nr4a1* mediated by NAC in the temporal window of 1 h may exert beneficial effects in the early response to novel challenges.

Despite the exact mechanism by which NAC enhances *Nr4a1* activity is not being fully understood, and more studies are needed to determine the magnitude of this interplay, our results suggest that enhancing *Nr4a1* by NAC may be a way to protect against oxidative stress and so counteract excessive ROS production, suggesting its therapeutic potential in a range of conditions associated with oxidative stress.

Moreover, recent studies reported that NAC improves memory impairments in animal models of schizophrenia [33] and epilepsy [34]. Actually, the role of acute stress in modulating memory and learning consolidation has been widely described [35] and the observation that NAC magnified the effect of the exposure to the acute challenge in comparison to VLX indicated that this compound has the potential to be further explored as a possible enhancer of cognitive abilities. In line with this, we recently found that, in the same experimental conditions, 1 h of ARS enhanced the cognitive performance in the novel object recognition test [11], suggesting that pretreatment with NAC may further improve the cognitive response by inducing the expression of *Nr4a1*.

Further support derived from the observation that, as mentioned above, the major contribution to the results obtained derives from the modulations of *Nr4a1* in the ventral hippocampus and amygdala, the brain regions actively involved in the mechanisms of learning and memory.

The overall findings of this work indicated that NAC could be useful to promote coping strategies to face not only an acute challenge but also, perhaps, persistent stressful events, factors primarily responsible for the development of stress-related disorders.

However, evidence exists that other antidepressants, such as vortioxetine [14] and fluoxetine [36], increased the expression of this gene, further supporting the notion that *Nr4a1* may represent a target for the development of novel drugs to treat stress-related disorders.

Further, the Z-activation score also indicated that NAC increased the expression of *Nr4a1,* whereas venlafaxine did not, in naïve animals adding critical information for the possible use of NAC in healthy subjects to promote active coping in, for instance, adverse situations or in physiological aging, known to be associated with neuronal oxidative stress and inflammation [37,38].

The fact that the study was conducted only in males is, indeed, one of the limitations of this work, so we will plan our future studies to also include females. Indeed, since depression occurs more in females than in males, the study in females will add critical information regarding the possibility of using NAC as a beneficial compound in both sexes. Moreover, we investigated the effect of chronic treatment with NAC and VLX on the response to the acute restraint stress at one single time point only. Given the dynamic nature of IEG responses, our analyses may not be complete; however, our preliminary results indicate that 1 h after ARS is the best timing to discriminate the expression of markers of neuroplastic mechanisms at the central level. The exploration of the time course of NAC’s effects following the ARS at different timings will be interesting to evaluate whether NAC may exert long-lasting effects both at the behavioral and molecular levels.

## 4. Materials and Methods

### 4.1. Animals

Adult male Wistar rats (5 weeks old) (Charles River, Sulzfeld, Germany) were brought into the laboratory two weeks before the start of the experiment. Animals were housed in standard laboratory conditions: food and water were freely available on a 12 h light/dark cycle, constant temperature (22  ±  2 °C) and humidity (50 ± 5%). All procedures used in this study have conformed to the rules and principles of the 86/609/EEC Directive and have been approved by the Local Bioethical Committee at the Maj Institute of Pharmacology, Polish Academy of Sciences, Krakow, Poland. All efforts were made to minimize animal suffering, to reduce the number of animals used and to ensure that the animal studies complied with the ARRIVE guidelines.

### 4.2. Pharmacological Treatment and Stress Procedure

Rats were treated with a daily intraperitoneal injection of saline (VEH) or venlafaxine HCl (VLX) at the dose of 10 mg/kg or N-acetylcysteine (NAC) at the dose of 300 mg/kg for 21 days. Twenty-four hours after the last drug administration, half of the animals were left undisturbed (Naïve) in their home cages while the other half were exposed to one hour of acute restraint stress (ARS). During the ARS, animals were placed into perforated plastic tubes (6.5 cm inner diameter) of adjustable length.

**Figure 6:**
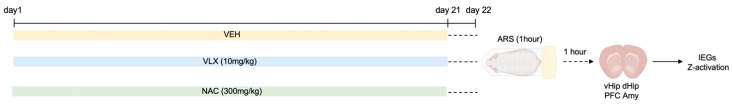
Schematic representation of the experimental paradigm.

Based on this experimental design depicted in Figure 6, we obtained the following 6 experimental groups, to which rats were randomly assigned (the researchers were blinded to the group allocation at any stage of the experiment and data analysis):

(1) VEH/Naïve: rats treated with VEH and not exposed to the ARS (n = 6).
(2) VEH/ARS: rats treated with VEH and exposed to the ARS (n = 6).
(3) VLX/Naïve: rats treated with VLX (10 mg/kg) and not exposed to the ARS (n = 6).
(4) VLX/ARS: rats treated with VLX (10 mg/kg) and exposed to the ARS (n = 6).
(5) NAC/Naïve: rats treated with NAC (300 mg/kg) and not exposed to the ARS (n = 6).
(6) NAC/ARS: rats treated with NAC (300 mg/kg) and exposed to the ARS (n = 6).

Rats were sacrificed 1 h after the end of the stress session and prefrontal cortex, amygdala and both dorsal and ventral hippocampi were dissected from the whole brain according to the plates of the atlas of Paxinos and Watson for the subsequent molecular analyses.

### 4.3. RNA Preparation and Gene Expression by Quantitative Real-Time PCR

Total RNA was isolated by a single step of guanidinium isothiocyanate/phenol extraction using PureZol RNA isolation reagent (Bio-Rad Laboratories, Segrate, Italy) according to the manufacturer’s instructions and quantified by spectrophotometric analysis. An aliquot of each sample was then treated with DNase (ThermoFisher Scientific, Monza, Italy) to avoid DNA contamination. The samples were processed for real-time polymerase chain reaction (RT-PCR) to assess *Arc*, *c-Fos*, *Gadd45β*, *Npas4*, *Nr4a1* and *Zif268* mRNA levels. RNA was analyzed by TaqMan qRTPCR instrument (CFX384 real time system, Bio-Rad Laboratories, Segrate, Italy) using the iScriptTM one-step RT-PCR kit for probes (Bio-Rad Laboratories, Segrate, Italy). Samples were run in 384-well formats in triplicate as multiplexed reactions with a normalizing internal control (*36B4*). A comparative cycle threshold (Ct) method was used to calculate the relative target gene expression. Primer sequences used were purchased from Eurofins MWG-Operon (Table 1) and Life Technologies (Table 2).

### 4.4. Z-Score

Z-score for each gene was calculated for each animal according to the following formula:

$$\mathrm{Z-score}=\frac{X-µ}{\sigma }$$

X represents the individual gene expression data for each animal while µ and σ represent the mean and the standard deviation for the VEH/Naïve group. Z-score activation was calculated by averaging the Z-score of each gene in all the brain regions.

### 4.5. Statistical Analysis

The molecular results were analyzed with the two-way analysis of variance (ANOVA) with stress and pharmacological treatments as independent factors (Tables S1 and S2). When appropriate, further differences were analyzed by Tukey’s multiple comparisons test. Significance for all tests was assumed for *p* < 0.05.

## 5. Conclusions

In conclusion, our findings point out that *Nr4a1* is a target to be further investigated in the context of resilience and may support the role of NAC as a putative compound for the amelioration of neuronal plasticity as well as for the enhancement of memory mechanisms and other functional abilities, which are deteriorated in patients affected by psychiatric disorders.

## Figures and Tables

**Table 1:** Sequences of forward and reverse primers and probes used in real-time PCR analyses and purchased from Eurofins MWG-Operon.

Gene	Forward Primer	Reverse Primer	Probe
*Arc*	GGTGGGTGGCTCTGAAGAAT	ACTCCACCCAGTTCTTCACC	GATCCAGAACCACATGAATGGG
*c-Fos*	TCCTTACGGACTCCCCAC	CTCCGTTTCTCTTCCTCTTCAG	TGCTCTACTTTGCCCCTTCTGCC
*Npas4*	TCATTGACCCTGCTGACCAT	AAGCACCAGTTTGTTGCCTG	TGATCGCCTTTTCCGTTGTC
*Zif268*	GAGCGAACAACCCTACGAG	GTATAGGTGATGGGAGGCAAC	TCTGAATAACGAGAAGGCGCTGGTG
*36B4*	TCAGTGCCTCACTCCATCAT	AGGAAGGCCTTGACCTTTTC	TGGATACAAAAGGGTCCTGG

**Table 2:** Probes purchased from Life Technologies which did not disclose the sequence.

Gene	Accession Number	Assay ID
*Gadd45β*	BC085337.1	Rn01452520_g1
*Nr4a1*	BC097313.1	Rn01533237_m1

## Data Availability

The data presented in this study are available on request from the corresponding author.

## Supplementary Materials

The following supporting information can be downloaded at https://www.mdpi.com/article/10.3390/ijms24087321/s1.
