# Supplementary material for: Chronic N-Acetyl-Cysteine Treatment Enhances the Expression of the Immediate Early Gene Nr4a1 in Response to an Acute Challenge in Male Rats: Comparison with the Antidepressant Venlafaxine
Source: Int J Mol Sci. 2023 Apr 15;24(8):7321. doi: 10.3390/ijms24087321 (PMC10139155; doi:10.3390/ijms24087321)
Supplement: Supplementary file 1 [file ijms-24-07321-s001.zip › ijms-2268907-supplementary.pdf]

	Two-way ANOVA	F (dFn, dFd)	P value
**vHip**			
Arc	treatment	F (2, 33) = 11.2	p=0.000
	challenge	F (1,34) =49.5	p=0.000
	treatment X challenge	F (2, 33) = 3.11	p=0.059
Cfos	treatment	F (2, 31) = 13	p=0.000
	challenge	F (1, 32) = 173	p=0.000
	treatment X challenge	F (2, 31) = 17.5	p=0.000
Npas4	treatment	F (2, 33) = 4.98	p=0.013
	challenge	F (1, 34) =0.646	p=0.421
	treatment X challenge	F (2, 33) = 2.76	p=0.080
Zif268	treatment	F (2, 32) = 17.1	p=0.000
	challenge	F (1, 33) = 34.8	p=0.000
	treatment X challenge	F (2, 32) = 2.67	p=0.087
Gadd45β	treatment	F (2, 33) = 16.7	p=0.000
	challenge	F (1, 34) = 45.2	p=0.000
	treatment X challenge	F (2, 33) = 0.16	p=0.845
Nr4a1	treatment	F (2, 34) = 16.9	p=0.000
	challenge	F (1, 35) = 22.1	p=0.000
	treatment X challenge	F (2, 34) = 1.57	p=0.224
**dHip**			
Arc	treatment	F (2, 34) = 4.47	p=0.020
	challenge	F (1, 35) = 19	p=0.000
	treatment X challenge	F (2, 34) = 2.99	p=0.065
Cfos	treatment	F (2, 33) = 8.18	p=0.001
	challenge	F (1, 34) = 244	p=0.000
	treatment X challenge	F (2, 33) = 4.97	p=0.014
Npas4	treatment	F (2, 34) = 9.34	p=0.000
	challenge	F (1, 35) = 0.43	p=0.516
	treatment X challenge	F (2, 34) = 2.98	p=0.065
Zif268	treatment	F (2, 33) = 4.92	p=0.014
	challenge	F (1, 34) =5.21	p=0.030
	treatment X challenge	F (2, 33) = 4.52	p=0.019
Gadd45β	treatment	F (2, 34) = 3.95	p=0.030
	challenge	F (1, 35) = 33.7	p=0.000
	treatment X challenge	F (2, 34) = 4.12	p=0.026
Nr4a1	treatment	F (2, 34) = 26.4	p=0.000
	challenge	F (1, 35) = 9.55	p=0.004
	treatment X challenge	F (2, 34) =2.80	p=0.076
**Pfc**			
Arc	treatment	F (2, 34) = 3.22	p=0.054
	challenge	F (1, 35) = 210	p=0.000
	treatment X challenge	F (2, 34) = 2.05	p=0.146
Cfos	treatment	F (2, 33) = 1.77	p=0.188
	challenge	F (1, 34) = 235	p=0.000
	treatment X challenge	F (2, 33) = 0.689	p=0.509
Npas4	treatment	F (2, 33) = 6.27	p=0.005
	challenge	F (1, 34) = 23	p=0.000
	treatment X challenge	F (2, 33) = 3.26	p=0.052
Zif268	treatment	F (2, 33) = 47.2	p=0.000
	challenge	F (1, 34) = 155	p=0.000
	treatment X challenge	F (2, 33) = 13.8	p=0.000

Gadd45β	treatment	F (2, 34) = 0.713	p=0.498
	challenge	F (1, 35) = 158	p=0.000
	treatment X challenge	F (2, 34) = 2.13	p=0.136
Nr4a1	treatment	F (2, 34) = 0.488	p=0.618
	challenge	F (1, 35) = 94.3	p=0.000
	treatment X challenge	F (2, 34) = 1.80	p=0.1833
**Amy**			
Arc	treatment	F (2, 34) = 4.95	p=0.013
	challenge	F (1, 35) = 129	p=0.000
	treatment X challenge	F (2, 34) = 7.18	p=0.002
Cfos	treatment	F (2, 32) = 3.91	p=0.031
	challenge	F (1, 33) = 170	p=0.000
	treatment X challenge	F (2, 32) = 6.77	p=0.004
Npas4	treatment	F (2, 33) = 1.05	p=0.364
	challenge	F (1, 34) = 4.96	p=0.033
	treatment X challenge	F (2, 33) = 2.82	p=0.074
Zif268	treatment	F (2, 34) =0.40	p=0.667
	challenge	F (1, 35) = 33.6	p=0.000
	treatment X challenge	F (2, 34) = 0.38	p=0.687
Gadd45β	treatment	F (2, 34) = 1.80	p=0.182
	challenge	F (1, 35) = 186	p=0.000
	treatment X challenge	F (2, 34) =2.58	p=0.092
Nr4a1	treatment	F (2, 33) = 2.58	p=0.092
	challenge	F (1, 34) = 93.7	p=0.000
	treatment X challenge	F (2, 33) = 2.72	p=0.828

Table S1: Two-way ANOVA analysis of mRNA levels in the ventral hippocampus (vHip), dorsal hippocampus (dHip), prefrontal cortex (Pfc) and amygdala (Amy) of rats treated with VLX (10mg/kg) and NAC (300mg/kg) and exposed to 1 hour of acute restraint stress (challenge).

IEGs Z-activation	Two-way ANOVA	F (dFn, dFd)	P value
Arc	treatment	F (2, 34) = 2.04	p=0.1478
	challenge	F (1, 35) = 183	p=0.000
	treatment X challenge	F (2, 34) = 3.67	p=0.037
c-Fos	treatment	F (2, 34) = 2.97	p=0.066
	challenge	F (1, 35) = 207	p=0.000
	treatment X challenge	F (2, 34) = 3.71	p=0.036
Npas4	treatment	F (2, 34) = 7.14	p=0.002
	challenge	F (1, 35) = 0.71	p=0.404
	treatment X challenge	F (2, 34) = 3.82	p=0.033
Zif268	treatment	F (2, 34) = 7.27	p=0.002
	challenge	F (1, 35) = 22.2	p=0.000
	treatment X challenge	F (2, 34) = 1.29	p=0.290
Gadd45β	treatment	F (2, 34) = 6.02	p=0.006
	challenge	F (1, 35) = 147	p=0.000
	treatment X challenge	F (2, 34) = 1.27	p=0.294
Nr4a1	treatment	F (2, 33) = 25.4	p=0.000
	challenge	F (1, 34) = 109	p=0.000
	treatment X challenge	F (2, 33) = 2.59	p=0.091

Table S2: Two-way ANOVA analysis of the Z activation IEGs in the four brain regions of rats treated with VLX (10mg/kg) and NAC (300mg/kg) and exposed to 1 hour of acute restraint stress (challenge).
